# Optimal control for disease vector management in SIT models: an integrodifference equation approach

**DOI:** 10.1007/s00285-019-01327-6

**Published:** 2019-02-07

**Authors:** Klodeta Kura, Doran Khamis, Claire El Mouden, Michael B. Bonsall

**Affiliations:** Mathematical Ecology Research Group, Department of Zoology, University of Oxford, Oxford, OX1 3PS UK

**Keywords:** Density dependence, Dispersal, Mosquito control, 92D25, 92D40, 92B05

## Abstract

Vector-borne diseases are a major public health concern inflicting high levels of disease morbidity and mortality. Vector control is one of the principal methods available to manage infectious disease burden. One approach, releasing modified vectors (such as sterile or GM mosquitoes) Into the wild population has been suggested as an effective method of vector control. However, the effects of dispersal and the spatial distribution of disease vectors (such as mosquitoes) remain poorly studied. Here, we develop a novel mathematical framework using an integrodifference equation (discrete in time and continuous in space) approach to understand the impact of releasing sterile insects into the wild population in a spatially explicit environment. We prove that an optimal release strategy exists and show how it may be characterized by defining a sensitivity variable and an adjoint system. Using simulations, we show that the optimal strategy depends on the spatially varying carrying capacity of the environment.

## Introduction

Vector borne diseases are a major public health concern, causing high levels of morbidity and leading to nearly one million deaths, annually (WHO 2016). Often vector control methods are the only feasible management option with the aim of vector control being to suppress or often eliminate insect vector populations. The sterile insect technique (SIT) is a well established empirical method for reducing population size. This technique has been widely used to suppress or eradicate many insect pest species, through the release of modified insects (lab-sterilised, irradiated and/or novel genetic technologies) (Alphey et al. 2010). Control is achieved as mating between sterile and wildtype mosquitoes reduces the reproductive potential of the wild population.

In evaluating the efficacy of SIT, mathematical modelling is an effective tool (Manoranjan and van den Driessche 1986; Isidoro et al. 2009; Yakob and Bonsall 2009; Legros et al. 2012; Li and Zou 2012; Potgieter et al. 2013; Li and Yuan 2015). SIT is an area-wide control method and hence involves spatial domains. Different continuous or discrete spatial model types have been proposed to investigate the effects of space on the efficacy of SIT. The choice between different model formulations depends on the specific biological problem (see Table 1). An early example is that developed by Manoranjan and van den Driessche (1986) who analysed the effect of non-uniform sterile insect releases in a heterogeneous environment. Another modelling approach explored considered random releases across the environment and concluded that the effectiveness of SIT is highly influenced by spatial heterogeneity (Ferreira et al. 2008). More recently, Yakob and Bonsall (2009) developed a spatially explicit model where the release of sterile insects was assumed to be uniform across space. Their research suggests that high levels of insect dispersal reduces the effectiveness of the SIT. Legros et al. (2012) analysed and compared two release strategies: (1) spatially uniform releases and (2) releases at discrete locations. They concluded that uniform releases are more effective than releasing at specific locations.

**Table 1:** Eclectic synopsis of different spatial mathematical models developed for understanding mosquito control using SIT methods

	Model assumption	Results
Manoranjan and van den Driessche (1986)	Modelled the effectiveness of SIT using a reaction–diffusion equation. Sterile mosquitoes do not disperse while the wild population disperse randomly. 2*n* releases per space unit and the sex ratio of sterile and wild mosquitoes is one-to-one	The number of releases required for population elimination depends on the growth parameters of mosquitoes, domain length and the initial population distribution
Ferreira et al. (2008)	Used a stochastic two-dimensional cellular automata (discrete time and space). Releases based on ratio between sites occupied by sterile and wild mosquitoes. The diffusion is incorporated using Margolus neighborhood	The wild population can not be eliminated for a spatially heterogeneous distribution of mosquitoes
Yakob and Bonsall (2009)	Developed a spatially explicit model to find the optimal timing and sex specificity of lethal transgene activation for the control of different types of pest population. Sterile mosquitoes are uniformly distributed across the space	Optimal release strategy is influenced by the growth of the population, mosquito stage structuring, competition, and space. High rates of mosquito dispersal reduces the effectiveness of SIT
Legros et al. (2012)	Applied a ‘Skeeter Buster’ model which is a stochastic, spatially explicit model of *Ae. aegypti*. Assumed the daily dispersal of mosquitoes to be limited to the nearest neighbours. Applicable to small-scale settings	Population elimination is feasible only in small geographical settings, unless the habitat and releases are homogeneous

However, a constant dilemma is whether model predictions replicate real biological situations as the field data are often incomplete or not straightforward to analyse. In order to achieve successful vector control, we need a much more thorough understanding of how mosquitoes would respond to these control interventions (such as sterile insect releases) by focusing more on their biology, ecology and behaviour (Alphey et al. 2010). Spatial spread is a key element in mosquito reproduction and some studies argue that SIT technique may not be very effective in controlling mosquito populations due to their dispersal and distribution (Ferreira et al. 2008).

To address this issue we present an alternative modelling framework that allows us to consider population dynamic outcomes associated with different dispersal behaviour. Integrodifference equations (IDEs), which are discrete in time and continuous in space, incorporate a dispersal kernel for the spatial distribution of mosquitoes, model populations in which growth and dispersal do not happen at the same time (Kot and Schaffer 1986; Hardin et al. 1990; Kot 1992; Neubert et al. 1995; Kot et al. 1996; Neubert and Parker 2004; Hsu and Zhao 2008; Zhou and Kot 2011; Reimer et al. 2016). Unlike the model approaches discussed in Table 1, IDEs allow a wide range of redistribution kernels for dispersal to be considered. This way of formulating time and space also provides a better approach for determining invasion speeds (compared to the reaction–diffusion PDE approach which can often underestimate patterns of spread; see Kendall 1965; Murray 2001).

The aim of this paper is to use optimal control theory to find the most efficient release strategy under different environmental conditions and dispersal behaviours. This is of critical importance as it can be used as a guideline for the number of sterile mosquitoes that need to be reared as well as aiding best practice for their release. Specifically, we develop a bioeconomic model with the corresponding cost function for the control of the wild mosquito population, where the control parameter *r* describes the release ratio that should be applied.

Previous work (Gaff et al. 2007; Joshi et al. 2007; Martinez et al. 2015) has considered optimal harvesting in an integrodifference population framework, but to the best of our knowledge no one so far has investigated the effectiveness of SIT through an IDE framework. In particular we will analyse the effect of spatial heterogeneity on the sterile release strategies. We find that an optimal control release strategy exists and efficiently suppresses the wild mosquito population. Our approach allows us to find the timing and the intensity of the control that needs to be applied. In Sect. 2 we introduce the integrodifference framework. We present the model in Sect. 3 and derive the characterization of the optimal control in Sect. 4. This characterization corresponds to the most cost-effective release strategy to suppress or eliminate the wild mosquito population. In Sect. 5 we use numerical simulations to illustrate the theory developed.

## Integrodifference equations

In order to familiarise the reader with the integrodifference equation (IDE) framework (Kot and Schaffer 1986; Hardin et al. 1990; Kot 1992; Neubert et al. 1995; Kot et al. 1996; Neubert and Parker 2004; Hsu and Zhao 2008; Zhou and Kot 2011; Reimer et al. 2016), we give some basic notations and assumptions. Let $N_t$ be the population density at time *t*, $f(N_t)$ the growth function where $f(N_t )=N_t g(N_t)$ and $g(N_t)$ is the per capita growth. We model the population (in discrete time) with no movement as

1 $$\begin{aligned} N_{t+1}=f(N_t )=N_{t} g(N_{t}), \end{aligned}$$

with initial condition $N_{t_{0}}=N_0$ for $N_0>0$. The spatial spread is described by a dispersal kernel, denoted by *k*(*x*, *y*), which is a probability density function (pdf) and gives the probability that an individual starting at point *y*, will settle at point *x* by the next time step. The number of individuals moving to location *x* is found by integrating the dispersal kernel *k*(*x*, *y*) over the domain of interest. Hence, we have $\int _{\Omega } k(x,y)dy\le 1$ (as we are looking at a population on a finite domain), where $\Omega$ is the spatial domain. We get the full dynamics of the population by combining the growth function with the dispersal kernel as follows:

2 $$\begin{aligned} N_{t+1}(x)=\int _{\Omega } k(x,y)f(N_t (y);y)dy. \end{aligned}$$

## The model

For the SIT control problem, we assume an unstructured mosquito population with no overlapping generations. Let $W_t$ denote the wild mosquito population at time t. In the absence of sterile mosquitoes and (initially) ignoring any spatial variation, the governing difference equation for the wild mosquito population is

3 $$\begin{aligned} W_{t+1}=\alpha \beta W_{t} \gamma (W_{t}), \end{aligned}$$

where $\alpha$ is the number of matings per individual and $\beta$ is the number of offspring produced per mating. $\gamma (W_{t})$ is the survival probability and we assume a Ricker-type nonlinearity, such that: $\gamma (W_{t})=e^{-d-KW_{t}}$. The death function is approximated by the linear function $d+KW_t$, where *d* is the density independent death rate, $e^{-d}$ is the density independent survival probability and *K* is related to the carrying capacity. Now define $A=\alpha \beta e^{-d}$ (as the intrinsic population growth rate) and assume that no mating difficulties arise. Therefore, Eq. (3) takes the form:

4 $$\begin{aligned} W_{t+1}=AW_t e^{-KW_t}. \end{aligned}$$

Suppose now that $R_t$ sterile mosquitoes are released at time *t* into the wild mosquito population. The total population size is then $N_t=W_t+R_t$. The number of offspring produced by a wild mosquito over its life that would make it to adulthood without density-dependence, given that random mating with sterile mosquitoes occurs is $\frac{AW_{t}}{W_{t}+R_{t}}$. We assume that the life cycle happens in one time unit and that density dependence occurs only at the larval stage (Clements 1992; Lord 1998). This means that the survival probability depends only on the number of wild mosquitoes (and is independent of $R_t$). We assume that the number of released mosquitoes is $R_t=r_{t}W^{*}$, where $r_t$ is the release ratio at time step *t* and $W^{*}$ is the equilibrium population; the release of sterile mosquitoes is proportional to the wildtype equilibrium population size. Under these assumptions the population dynamics in a non-spatial system are governed by the following equation:

5 $$\begin{aligned} W_{t+1}=\frac{1}{1+r_{t}\frac{W^{*}}{W_{t}}}AW_{t}e^{-KW_{t}}. \end{aligned}$$

Next, we introduce spatial effects by incorporating the dispersal kernels and growth functions into our IDE framework. Assume that the wild mosquitoes have a dispersal kernel *k*(*x*, *y*) in a one dimensional domain $\Omega$. We assume a closed domain, for example an area that is surrounded by unfavourable conditions such that mosquitoes would not travel across. It is important to note that this might not always be the case and different spatial analyses will be needed for these different boundary conditions. To analyse the dynamics of the wild mosquito population under the influence of SIT release, we use:

6 $$\begin{aligned} W_{t+1}(x)= \int _{\Omega }\frac{1}{1+r_{t}(y)\frac{W^{*}}{W_{t}}} k(x,y)f(W_t (y),y)dy, \end{aligned}$$

where $f(W_t (y),y)=A W_{t}(y) e^{-KW_t(y)}$ and initial condition $W_0$ (Table 2). The equilibrium population $W^{*}$ is calculated as $W^{*}=Sf(W^{*})$ where $f(W^{*})$ is the growth function and *S* is the average dispersal success over the domain $\Omega$, given by $S=\dfrac{1}{\Omega }\int _{\Omega }\int _{\Omega }k(x,y)dx dy$. See van Kirk and Lewis (1997) and Reimer et al. (2016) for a more detailed analysis on equilibrium solutions.

**Table 2:** Description of the variables and parameters of the IDE model

Variable	Description
$W_t$	Wild mosquito population at time *t*
$R_t$	Released mosquitoes at time *t*
$W^{*}$	Equilibrium for the wild mosquito population

Parameter	Description
$\alpha$	Number of matings per individual
$\beta$	Number of offspring produced per mating
$\gamma (W_t)$	Survival probability
*d*	Density independent death rate
*K*	Strength of negative feedback–proxy for the carrying capacity
*A*	Intrinsic population growth rate
$r_{t}(x)$	Release ratio at time step *t* and location *x*

Parameter values are given in the figure legends and associated text

## Optimal control formulation of integrodifference equation

In this section, we describe the optimal control framework for minimizing the cost of controlling the wildtype mosquito population. We propose to control a vector population over a time period $t\in [0,T]$. The state variable is $W(x)=(W_{0}(x), W_{1}(x),\ldots ,W_{T}(x))$ and the control is $r(x)=(r_{0}(x), r_{1}(x),\ldots , r_{T-1}(x))$, representing the wild mosquito population and the sterile insect release ratio respectively at location *x* and time step *t*, where the initial distribution $W_{0}(x)$ is given.

The kernels are bounded and measurable such that $|\int _{\Omega } k(x,y) dy |\le 1, \forall x\in \Omega$ and $0\le k(x,y)\le k_{1}$ for $(x,y)\in \Omega \times \Omega$, where $k_1$ is a constant. We assume that the function *f* is twice differentiable in $W_{t}(y)$ and that partial derivatives $\frac{\partial f(W)}{\partial W}$, $\frac{\partial ^{2} f(W)}{\partial W}$ are $L^{\infty }$ bounded for any $W\in L^{\infty }(\Omega )$. We want to find the optimal strategy that suppresses the mosquito population and minimizes the cost of vector control via the release of sterile mosquitoes. Assume there is a linear cost associated with the wild mosquitoes (due to impact on human health, lost tourism, etc.) which we denote by $m_t$. There is also a cost for producing and releasing sterile mosquitoes and we assume it to be a quadratic of the form,$( n_t r_{t}W^{*}+s_t r_{t}^{2}(W^{*})^{2})$. The cost function is a nonlinear relationship in r*. This choice of cost function is based on the reality that sterile insect releases, through mating disruption, introduce an Allee effect into the wild population (Bonsall et al. 2010; also see Kirschner et al. 1997 for similar choice of quadratic costs for a HIV chemotherapy application). Using these costs, we define the objective functional:

7 $$\begin{aligned} J(r)=\sum _{t=0}^{T-1} \int _{\Omega } \left[ m_{t}W_{t}(x)+n_{t}r_{t}(x)W^{*}(x)+s_{t}r_{t}^{2}(x)(W^{*})^{2}(x)\right] dx, \end{aligned}$$

with set of bounded controls given by

8 $$\begin{aligned} \Delta =\left\{ (r_{0} (x),\ldots ,r_{T} (x))\in (L^{\infty }(\Omega ))^{T} |0\le r_{k}(x)\le r_{max},t=0,1,\ldots ,T-1 \right\} . \nonumber \\ \end{aligned}$$

Our goal is to minimize the total cost of mosquito management by finding $r^{*}$ such that:

9 $$\begin{aligned} J(r^{*})=\min _{r\ge 0} J(r). \end{aligned}$$

In order to achieve this, we need to link the sensitivity $\psi _{t}(x)$ and adjoint system (append the difference equations (Eq. 6) to the objective functional to be minimized), to characterize the optimal control. By differentiating the state equation we show that the sensitivity satisfies:

$$\begin{aligned} \begin{aligned} \psi _{t+1}(x)&=\int _{\Omega }\frac{1}{1+r_{t}(y)\frac{W^{*}}{W_{t}}} k(x,y)\frac{\partial f(W_{t}(y),y)}{\partial W} \psi _{t}(y)dy\\&\quad -\,\int _{\Omega } \bigg (\frac{1}{1+r_{t}(y)\frac{W^{*}}{W_{t}}}\bigg )^{2} l_{t}(y) k(x,y)f(W_t (y),y)dy, \end{aligned} \end{aligned}$$

for t $$=$$ 0,1,2,...,T − 1

10 $$\begin{aligned} \psi _{0}(x)=0. \end{aligned}$$

Proof of this result is given in the “Appendix”. Using optimal control theory, we derive the adjoint system and the resulting optimal release strategy in the following theorem.

### Theorem 1

Given an optimal control $r^{*}$ and corresponding state solution $W^{*}=W(r^{*})$, there exist a weak solution $\lambda \in (L^{\infty }(\Omega ))^{T}$ satisfying the adjoint system

$$\begin{aligned} \lambda _{t}(x)=\frac{1}{1+r_{t}(x)\frac{W^{*}(x)}{W_{t}(x)}}\frac{\partial f(W_{t}(x),x)}{\partial W} \int _{\Omega } k(y,x)\lambda _{t+1}(y)dy+m_{t},\\ \end{aligned}$$

with the transversality condition

11 $$\begin{aligned} \lambda _{T}(x)=0, \end{aligned}$$

and furthermore

12 $$\begin{aligned} r_{t}^{*}(x)=\frac{(X-1)W_{t}(x)}{W^{^{*}}(x)}, \end{aligned}$$

where *X* is the solution of

13 $$\begin{aligned} X^{3}+bX^{2}+d=0 \end{aligned}$$

and *b* and *d* are defined by: $b=\frac{n}{2sW}-1$, $d=\frac{-f(W_t (x),x)\int _{\Omega } \lambda _{t+1}(y) k(y,x)dy}{2sW_{t}W^{*}}$.

Proof of this Theorem is given in the “Appendix”. We find the characterization of the optimal control by solving Eq. (13). Using the proof of Theorem 1 (see “Appendix”), if we analyse Eq. (32), we find its derivative is positive for all time. This implies that Eq. (32) has precisely one real root. If we obtain a negative real root, we set the control to zero. The solution is zero when $C>A_{1}$ indicating that the optimal strategy is to not release any sterile mosquitoes. This means that the burden imposed by the wild mosquito population (in terms of disease or nuisance biting) is so low that we do not need to instigate a sterile mosquito release programme. When the real root is positive, we release sterile insects at the release ratio dictated by the root.

## Numerical results

We use a forward-backward sweep numerical method (e.g., Lenhart and Workman 2007) to find the optimal control $r\in \Delta$, where

$$\begin{aligned} \Delta =\left\{ (r_{0} (x),\ldots ,r_{T} (x))\in (L^{\infty }(\Omega ))^{T} |0\le r_{k}(x)\le r_{max},t=0,1,\ldots ,T \right\} \end{aligned}$$

such that $J(r^{*})=min_{r\in \Delta }J(r)$. This iterative process starts by guessing the control (in our case we start with $r=0$), and using the initial conditions of the state we solve the state equation forward in time. The next step is to use the new values of the state to solve the adjoint equation backwards in time. The values of the adjoint equation are used to solve the characterization of the control in order to get a new control value at each time step. To speed up the convergence, the value of the control is updated by taking a convex combination of the new and old control value. The new calculations of the optimal control and states are compared with results from the previous iterations. If the difference between iterations is less than 1$\%$, the final control *r* is considered to be optimal.

### Homogeneous environments

Here, we apply the optimal control theory to homogeneous environment. To replicate such environments we assume that the value of *K* (related to the carrying capacity) is the same across the whole domain $\Omega$. Throughout this section, we assume a uniformly distributed wild population at $t=0$ with $m=n=1$, $s=5$ and consider $\Omega =[0,1]$, unless otherwise stated. We have chosen these values to correspond to a high cost of producing and releasing sterile insects compared to the cost associated with the wild mosquitoes. With this set of costs, we do not expect a complete elimination of the wild population. In the following, we investigate the Laplace kernel for the dispersal, which is described by:

14 $$\begin{aligned} k(x,y)=\sqrt{\frac{\kappa _L}{4D_L}} e^{\left( -|x-y|\sqrt{\frac{\kappa _L}{4D_L}}\right) } \end{aligned}$$

where the parameters associated with this kernel follow $\kappa _L=D_L=1$.

**Fig. 1:**
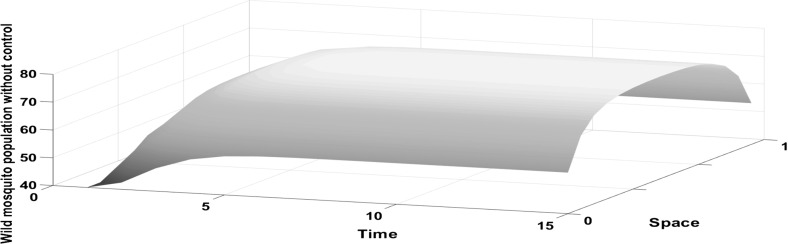
The density of the wild mosquito population without control increases initially until it approaches the equilibrium at time step $t=5$. A Laplace kernel is assumed for the dispersal in the domain $\Omega =[0,1]$ for $A=2$ and $K=0.001$

We have chosen an exponential form for the dispersal kernel, as these dispersal conditions have received the most support from the available data (Gratton and Zanden 2009; Estep et al. 2014). Model simulations show that in the absence of control, the wild mosquito population increases until it approaches the stable equilibrium at time step $t=5$ (see Fig. 1). The distribution of the wild mosquito population changes through time. Initially it has a uniform distribution, and as a result of their redistribution and the chosen boundary conditions, mosquitoes tend to aggregate more in the centre of the domain as opposed to its boundaries.

**Fig. 2:**
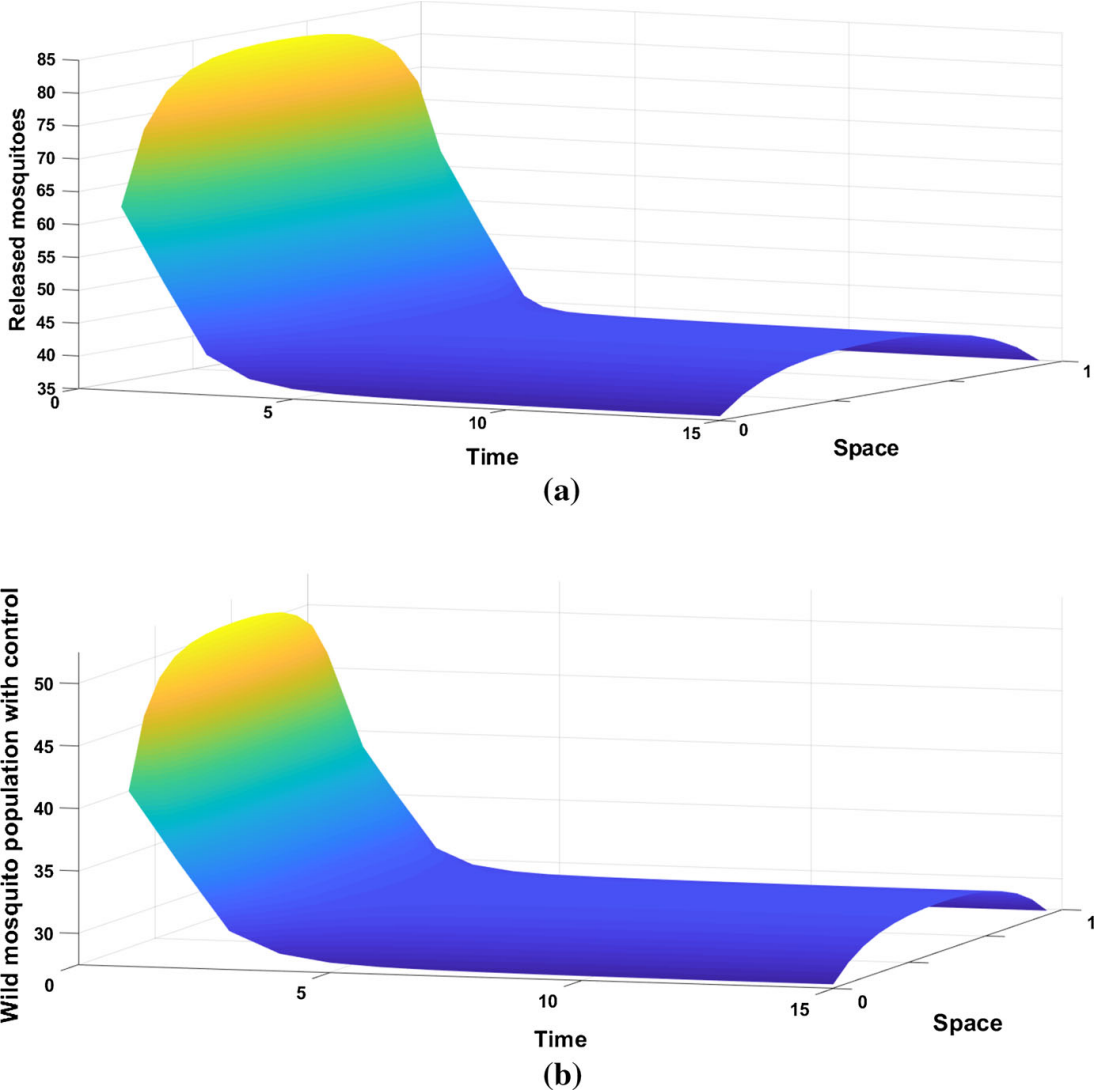
**a** The number of sterile mosquitoes released over time in a homogeneous environment. **b** The density of the wild mosquito population under the optimal control strategy in the domain $\Omega =[0,1]$ for $A=2$ and $K=0.001$. A Laplace kernel is assumed for the dispersal. Releasing sterile mosquitoes does not eliminate the wild population, but it significantly reduces it

When the control is introduced, the wild mosquito population decreases, but does not go to zero (see Fig. 2b). Similar to the behaviour without control, the population is aggregated in the centre of the domain and reduces towards the boundaries. For an effective SIT technique (capable of suppressing the wild mosquito population for the cost function and parameters given above), we need approximately 1.23:1 ratio of sterile to wild mosquitoes initially and releases should be applied more in the centre of the domain. The number of releases decreases through time (Fig. 2a) as the wild mosquito population size decreases to the point that they do not impose a high burden to the environment. This is the reason why the wild population size does not go to zero in Fig. 2b. Releasing sterile mosquitoes can reduce significantly the wild mosquito population, (by approximately $73\%$ by time step $t=5$) which could have a positive impact on disease management.

**Fig. 3:**
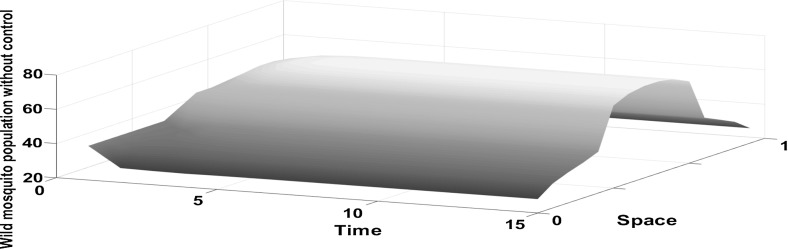
The density of the wild mosquito population increases at the centre of the domain. Wild mosquito population through time, with no control, when only the centre of the domain has favourable conditions. Laplace kernel is assumed

**Fig. 4:**
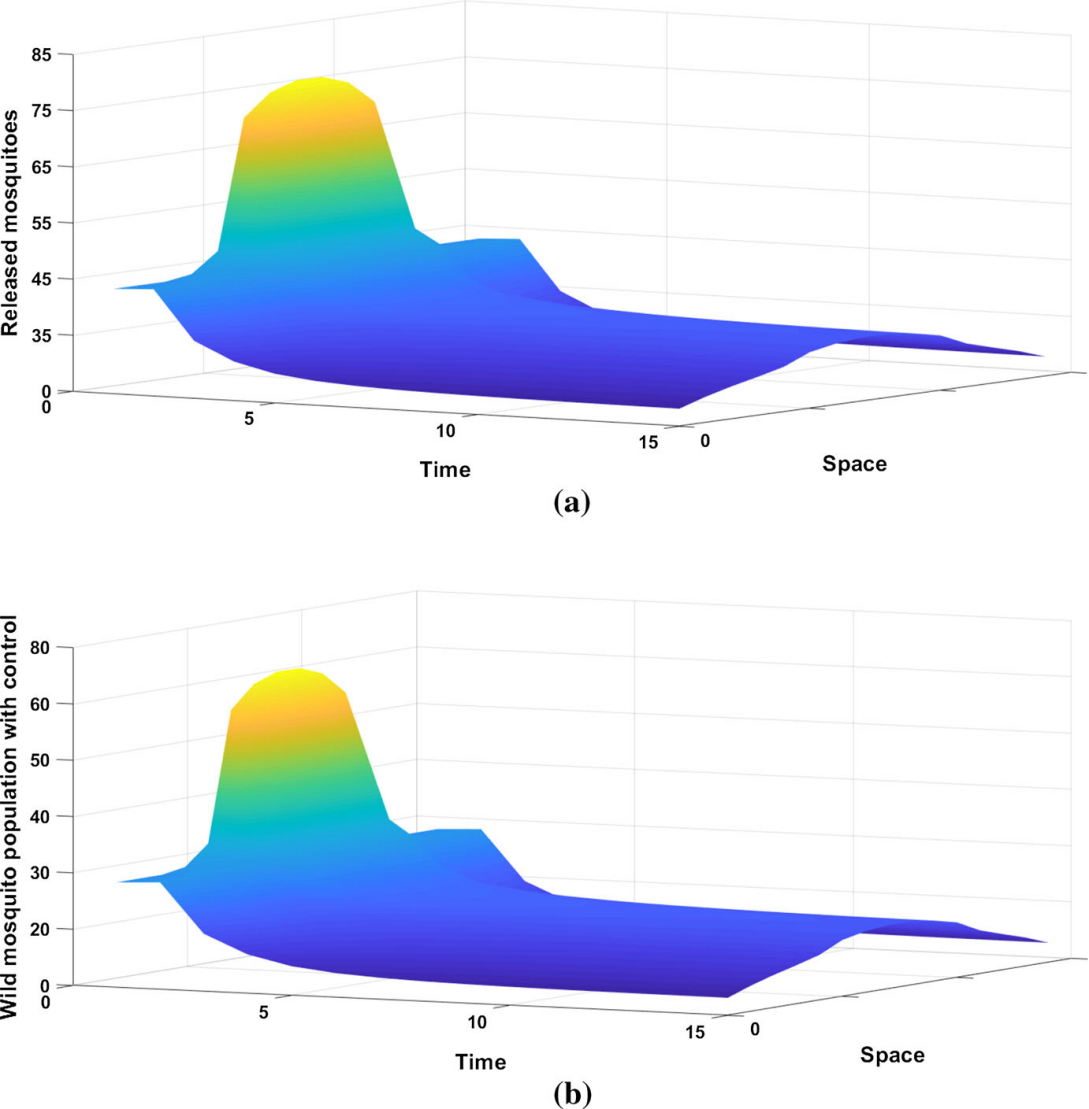
**a** The number of sterile mosquitoes released over time in the heterogeneous environment. **b** The density of the wild mosquito population under the optimal control strategy in the domain $\Omega =[0,1]$ for $A=2$ and $K=0.001$, when only the centre of the domain has favourable conditions. A Laplace kernel is assumed for the dispersal. Releasing sterile mosquitoes does not eliminate the wild population, but it significantly reduces it

**Fig. 5:**
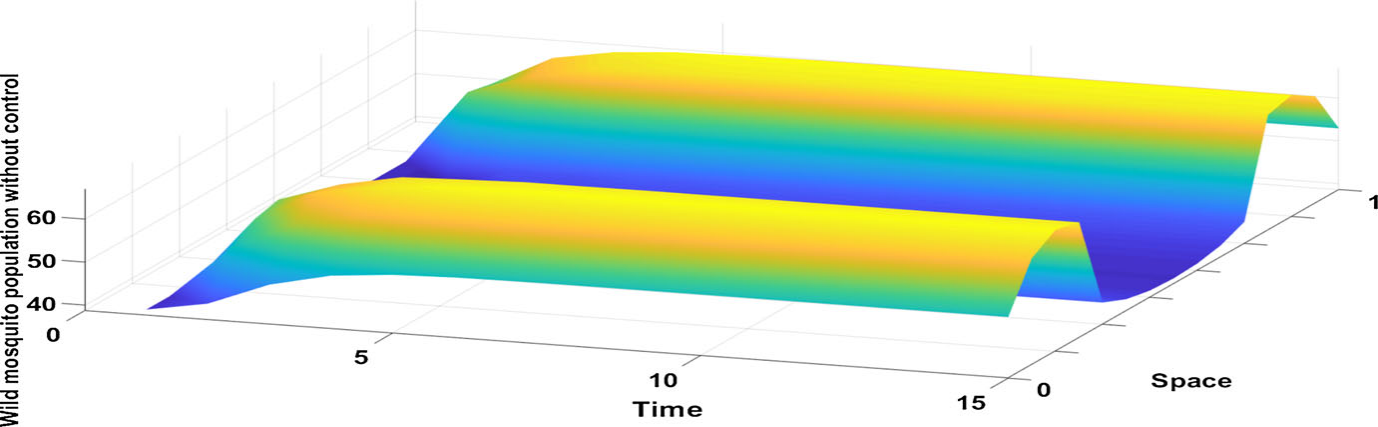
In the absence of control, the density of the wild mosquito population increases initially at the boundaries of the domain until it approaches the equilibrium

**Fig. 6:**
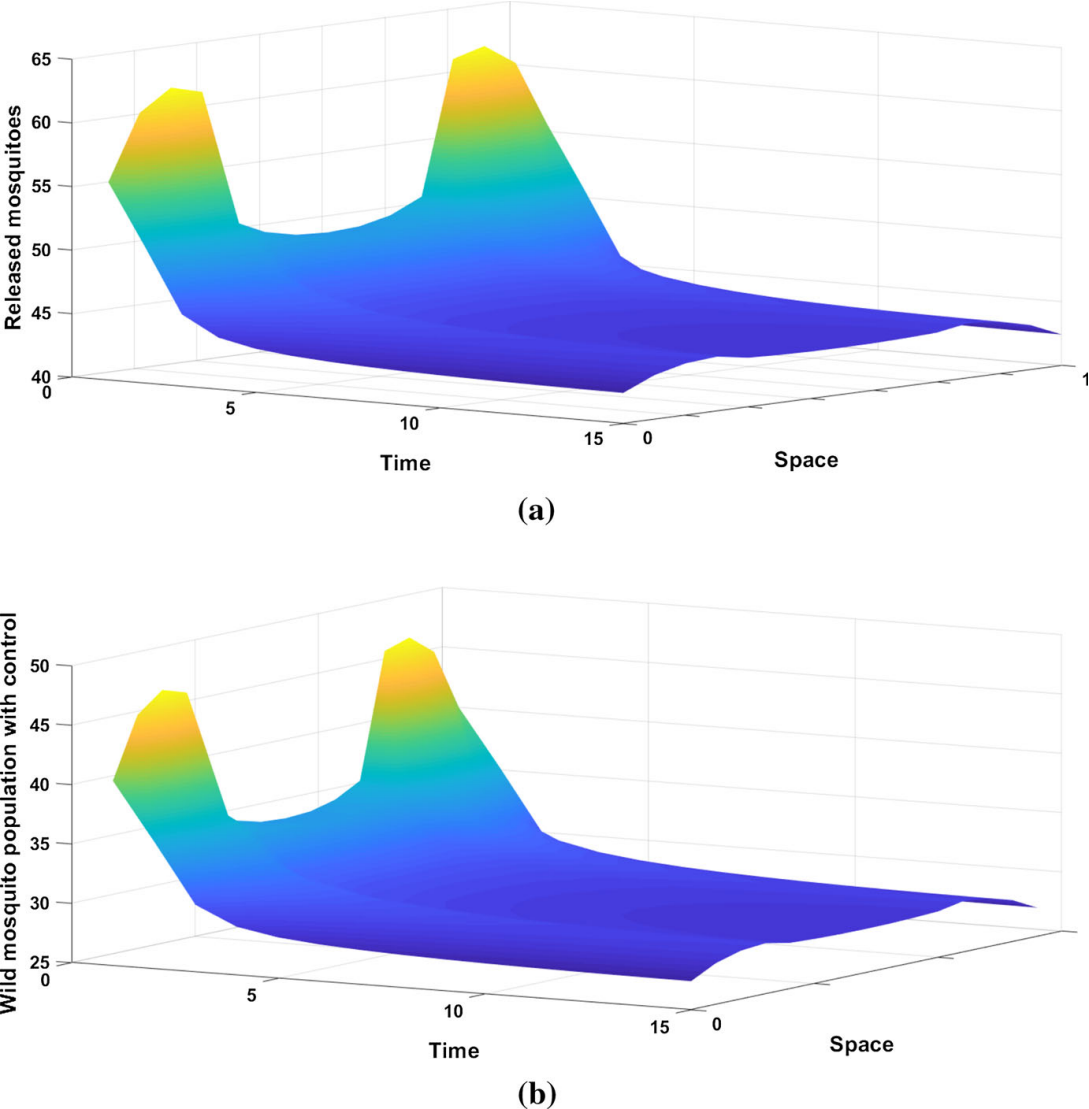
**a** The number of sterile mosquitoes released over time in the heterogeneous environment. **b** The density of the wild mosquito population under the optimal control strategy in the domain $\Omega =[0,1]$ for $A=2$ and $K=0.001$, when only the boundaries of the domain have favourable conditions. A Laplace kernel is assumed for the dispersal. Releasing sterile mosquitoes does not eliminate the wild population, but it significantly reduces it

### Heterogeneous environments

Very often, a habitat has different attractiveness to mosquitoes in different areas. This can be influenced by factors such as resource availability (e.g. food, mates, breeding sites) and predation. In this section, we analyse how landscape heterogeneity affects the dispersal and the optimal control of mosquitoes. We replicate this behaviour by varying the value of *K* (the strength of density feedbacks related to the carrying capacity). We consider situations (a) when the centre of the domain has more favourable Conditions (see Figs. 3, 4a), (b) when the boundaries of the domain have more favourable conditions (see Figs. 5, 6a). We conclude that through time, the population in situation (a) aggregates more in the centre of the domain, as expected, because the conditions are more favourable there. This is true for populations with and without control. Without control, the wild mosquito population slightly decreases initially at the boundaries until it approaches the stable equilibrium. On the other hand, in the centre of the domain, the wild mosquito population increases until it approaches its equilibrium at $t=5$. Once the control is introduced, we see from Fig. 4b that releasing sterile mosquitoes greatly suppresses the wild mosquito population. The optimal strategy in this scenario is to release more sterile mosquitoes in the centre of the domain where they are concentrated. Figure 4a gives the optimal number of sterile mosquitoes that need to be released in order to control the wild mosquito population. We release substantially more mosquitoes at the centre of the domain than its boundaries at time step $t=1$. This is because dispersing mosquitoes are moving more towards the centre and at this level the burden imposed by the wild mosquito population is so high that a large number of mosquitoes need to be released in order to suppress the population. Once the population is suppressed, after $t=2$, the ratio of releases between boundaries and centre of domain is decreased. We get the opposite behaviour when the conditions are more favourable in the boundaries. Without control, from Fig. 5, we notice that the population increases initially and it approaches an equilibrium at time $t=5$. As expected, the population size increases more in the boundaries, because mosquitoes will move more towards them as the conditions for mosquito reproduction are better. Using the control, we see from Fig. 6b that the population quickly decreases until it reaches a threshold where they do not impose a high burden. The optimal strategy in this case is to release more sterile insects in the boundaries initially (until $t=3$) and after the wild population has reached a critical low threshold (e.g., where the effects are not very harmful), the ratio of release between boundaries and the centre of domain is decreased.

## Discussion

Here, we formulated a novel mathematical model to understand the effects of releasing sterile mosquitoes into wild populations as well as the effect of spatial spread on mosquito population dynamics. The approach described here has not been used before in designing and optimizing sterile insect release strategies.

The model is described by an integrodifference equation, which are used to model populations where growth and dispersal do not happen at the same time. In our model we consider a homogeneous population consisting of wild and sterile mosquitoes with no overlapping generations. The growth function is based on the Ricker model and we assume releases proportional to wild population equilibrium. The spatial spread of the mosquitoes is described by the dispersal kernel *k*(*x*, *y*) in the integrodifference equation.

Using numerical simulations, we considered homogeneous environments with uniform carrying capacity, and heterogeneous environments with different carrying capacity in different areas, where the Laplace kernel describes mosquito movement. One significant finding is that, due to redistribution, applying the optimal control does not eradicate the wildtype mosquito but only substantially reduces population size. In practice, sterile mosquitoes may be released from aircraft resulting in releases that are approximately uniform. Our results highlight that this is not optimal and instead releasing more where the population densities are higher is more efficacious for vector control. Our model predicts a 73$\%$ suppression of the wild population which is close to observed field estimates, where $> 80\%$ suppression rates have been reported for *A. aegypti* control (Harris et al. 2012; Carvalho et al. 2015).

In heterogeneous environments, we consider situations where the centre of the domain has more favourable conditions or when the boundaries have more favourable conditions. In both cases the control significantly suppresses the mosquito population. Our results suggest (as expected) that more mosquitoes should be released where densities are higher. Another (expected) result from this model is that the optimal strategy is to release significantly more sterile mosquitoes at the beginning of the vector management control programme until the wild mosquito population is suppressed to a level that the imposed burden is so low.

Here we show that continuous releases predict that complete eradication of the wild population is not an optimal solution. Furthermore, integrating different control options (insecticide knockdowns, pulse or continuous SIT) to achieve cost-efficient control strategies needs more thorough investigation (Hackett and Bonsall 2018). Additionally, we argue that this discrete-time, continuous-space model approach is better than previous ones (described in Introduction) in finding the time and intensity of control, as it can include a variety of dispersal behaviour. This is crucial when modelling mosquito control. However, we emphasize the importance of determining a more accurate dispersal kernel that supports the field data, as the wave speed is very sensitive to the dispersal behaviour. Depending on the specific system parameterization, the results presented here are likely to be sensitive to the cost function form and the parameters used in this function. We have assumed a quadratic form, but other functions can be explored (see Khamis et al. 2018). This will change the objective functional *J*(*r*) and the characterization of the optimal control. Importantly, our results emphasize that optimal control does not necessarily lead to population elimination. Varying the parameters (*m*, *n*, & *s* in Eq. 7) associated with the cost of the wild and sterile mosquitoes will most likely modify this outcome. If we lower the cost of producing and releasing sterile mosquitoes, we can achieve elimination of the wild population. In summary, we showed that the optimal control of the SIT model described by an integrodifference equation exists and that the control can significantly suppress the wild mosquito population.

## Appendix

### Derivation of the sensitivity of the state with respect to the control

To characterize an optimal control we need to differentiate the map $r \longrightarrow J(r)$. In order to do this we need to first differentiate the control-to-solution map $r\longrightarrow W=W(r)$. The directional derivative $\psi$ of this solution map is known as the sensitivity of the state with respect to the control. The next step is to derive the equation of $\psi$ by taking the derivative of the control-to-solution map in the direction *l*, and assuming that the perturbed control function contains $W_{t}^{\epsilon } \approx W_{t}$.

#### Theorem 2

The mapping $r\longrightarrow W$ is differentiable in the following sense: for any $r\in \Delta$ and $l\in (L^{\infty }(\Omega ))^{T}$, such that $(r + \epsilon l)\in \Delta$ for small $\epsilon$, there exist sensitivity $\psi (x)\in (L^{\infty }(\Omega ))^{T+1}$ such that for $t=0,1,\ldots ,T$ we have $\frac{W_{t}^{\epsilon }(x)-W_{t}(x)}{\epsilon }\longrightarrow \psi _{t}(x)$ pointwise on $\Omega$. Furthermore the sensitivity satisfies:

15 $$\begin{aligned}&\psi _{t+1}(x)=\int _{\Omega }\frac{1}{1+r_{t}(y)\frac{W^{*}}{W_{t}}} k(x,y)\frac{\partial f(W_{t}(y),y)}{\partial W} \psi _{t}(y)dy \end{aligned}$$

16 $$\begin{aligned}&\qquad \qquad \qquad -\int _{\Omega } \left( \frac{1}{1+r_{t}(y)\frac{W^{*}}{W_{t}}}\right) ^{2} l_{t}(y) k(x,y)f(W_t (y),y)dy, \end{aligned}$$

for t $$=$$ 0,1,2,...,T − 1

17 $$\begin{aligned} \psi _{0}(x)=0 \end{aligned}$$

#### Proof

Consider the control-to-solution maps

18 $$\begin{aligned}&r \longrightarrow W=W(r) \nonumber \\&r+\epsilon l\longrightarrow W^{\epsilon }=W(r+\epsilon l) \end{aligned}$$

where *l* is an arbitrary variation. From Eqs. (6) and (18) we can write the following

$$\begin{aligned} W_{t+1}^{\epsilon }(x)=\int _{\Omega }\frac{1}{1+r_{t}(y)\frac{W^{*}}{W_{t}}}\left( 1-\frac{1}{1+r_{t}(y)\frac{W^{*}}{W_{t}}} \epsilon l_{t}(y)\frac{W^{*}(y)}{W_{t}(y)}\right) k(x,y)f(W_{t}^{\epsilon } (y),y)dy \end{aligned}$$

and obtain

19 $$\begin{aligned} \frac{W_{t+1}^{\epsilon }(x)-W_{t+1}(x)}{\epsilon }= & {} \int _{\Omega }\frac{1}{1+r_{t}(y)\frac{W^{*}}{W_{t}}} k(x,y)\frac{f(W_{t}^{\epsilon }(y),y)-f(W_{t}(y),y)}{\epsilon }dy\nonumber \\&\quad -\,\int _{\Omega }\left( \frac{1}{1+r_{t}(y)\frac{W^{*}}{W_{t}}}\right) ^{2} l_{t}(y)\frac{W^{*}(y)}{W_{t}(y)} k(x,y)f(W_{t}^{\epsilon } (y),y)dy\nonumber \\ \end{aligned}$$

Using $W_{0}^{\epsilon }=W_{0}$ we obtain

20 $$\begin{aligned} \frac{W_{1}^{\epsilon }(x)-W_{1}(x)}{\epsilon } = -\int _{\Omega }\left( \frac{1}{1+r_{0}(y)\frac{W^{*}}{W_{t}}}\right) ^{2} l_{0}(y)\frac{W^{*}(y)}{W_{0}(y)} k(x,y)f(W_{0}^{\epsilon } (y),y)dy\nonumber \\ \end{aligned}$$

This means that

21 $$\begin{aligned} \left| \frac{W_{1}^{\epsilon }(x)-W_{1}(x)}{\epsilon } \right| \le C_{1}, \forall x\in \Omega \end{aligned}$$

We notice from Eq. (20) that $\frac{W_{1}^{\epsilon }(x)-W_{1}(x)}{\epsilon }$ does not depend on $\epsilon$ and therefore $\frac{W_{1}^{\epsilon }(x)-W_{1}(x)}{\epsilon } \longrightarrow \psi _{1}(x)$ pointwise. Similarly we obtain $\frac{W_{2}^{\epsilon }(x)-W_{2}(x)}{\epsilon } \longrightarrow \psi _{2}(x)$. By iteration we have

22 $$\begin{aligned} \left| \frac{W_{t}^{\epsilon }(x)-W_{t}(x)}{\epsilon } \right| \le C_{t}, \forall x\in \Omega \end{aligned}$$

Subsequently we have $\frac{W_{t}^{\epsilon }(x)-W_{t}(x)}{\epsilon }\longrightarrow \psi _{t}(x)$ weakly in $L^{2}(\Omega )$. Using the pointwise convergence we can show that $\psi$ satisfies

23 $$\begin{aligned} \psi _{t+1}(x)= & {} \int _{\Omega }\frac{1}{1+r_{t}(y)\frac{W^{*}}{W_{t}}} k(x,y)\frac{\partial f(W_{t}(y),y)}{\partial W} \psi _{t}(y)dy \nonumber \\&\quad -\,\int _{\Omega } \left( \frac{1}{1+r_{t}(y)\frac{W^{*}}{W_{t}}}\right) ^{2} l_{t}(y) k(x,y)f(W_t (y),y)dy, \end{aligned}$$

$$\square $$

### Proof of Theorem 1

Let $r^{*}$ be an optimal control and $W(r^{*})$ the corresponding state. For the arbitrary variation *l* with $(r^{*}+\epsilon l)\in \Delta$ for very small positive $\epsilon$, let $W^{\epsilon }$ be the corresponding solution of the state Eq. (6). As the adjoint equation is linear in $\lambda$, there exists a weak solution $\lambda$ which satisfies Eq. (11). To characterize the optimal solution, we take the directional derivative of the cost function *J*(*r*) with respect to *r* in the direction *l*. Since $J(r^{*})$ is a minimum value we have

24 $$\begin{aligned}&0\le \lim _{\epsilon \rightarrow 0^{+}} \frac{J(r^{*}+\epsilon l)-J(r^{*})}{\epsilon } \nonumber \\&\quad =\lim _{\epsilon \rightarrow 0^{+}}\frac{1}{\epsilon } \left[ \sum _{t=0}^{T-1}\int _{\Omega } \left( m_{t}W_{t}^{\epsilon }(x)+n_{t} \left( r_{t}^{*}(x)+\epsilon l_{t}(x)\right) W^{*}(x)+s_{t}(r_{t}^{*}(x) \right. \right. \nonumber \\&\left. \qquad +\,\epsilon l_{t}(x))^{2}\right) (W^{*})^{2}(x)dx \nonumber \\&\quad \left. -\,\sum _{t=0}^{T-1}\int _{\Omega } (m_{t}W_{t}^{*}(x)+n_{t}r_{t}^{*}(x) W^{*}(x)+s_{t}(r_{t}^{*})^{2}(x)(W^{*})^{2}(x))dx\right] \nonumber \\&\quad =\sum _{t=0}^{T-1}\int _{\Omega } \left[ m_{t}\psi _{t}(x)+n_{t}W^{*}(x)l_{t}(x)+2s_{t}r_{t}^{*}l_{t}(x)(W^{*})^{2}(x) \right] dx \end{aligned}$$

In the last expression we have used $\frac{W_{t}^{\epsilon }(x)-W_{t}(x)}{\epsilon }= \psi _{t}(x)$ [from Eq. (22)]. We calculate first the term $\sum _{t=0}^{T-1}\int _{\Omega }\left[ m_{t}\psi _{t}(x) \right] dx$ by adding $\lambda _{T}\psi _{T}(x)$ (which is zero due to the transversality condition) and using the adjoint equation:

$$\begin{aligned}&\sum _{t=0}^{T-1}\int _{\Omega }\left[ m_{t}\psi _{t}(x) \right] dx =\sum _{t=0}^{T-1}\int _{\Omega }\left[ \lambda _{t}(x)-\frac{1}{1+r_{t}(x)\frac{W^{*}(x)}{W_{t}(x)}}\frac{\partial f(W_{t}(x),x)}{\partial W} \int _{\Omega } k(y,x)\lambda _{t+1}(y)dy \right] \psi _{t}(x)dx\\&\quad =\sum _{t=0}^{T-1}\int _{\Omega } \lambda _{t}(x)\psi _{t}(x)dx-\sum _{t=0}^{T-1}\int _{\Omega }\frac{1}{1+r_{t}(x)\frac{W^{*}(x)}{W_{t}(x)}}\frac{\partial f(W_{t}(x),x)}{\partial W} \psi _{t}(x) \int _{\Omega }k(y,x)\lambda _{t+1}(y)dydx\\&\quad =\sum _{t=0}^{T-1}\int _{\Omega } \left[ \lambda _{t}(x)\psi _{t}(x)+\lambda _{T}(x)\psi _{T}(x)\right] dx\\&\qquad -\sum _{t=0}^{T-1}\int _{\Omega }\frac{1}{1+r_{t}(x)\frac{W^{*}(x)}{W_{t}(x)}}\frac{\partial f(W_{t}(x),x)}{\partial W} \psi _{t}(x) \int _{\Omega }k(y,x)\lambda _{t+1}(y)dydx\\&\quad =\sum _{t=0}^{T-1}\int _{\Omega }\lambda _{t+1}(x)\psi _{t+1}(x)dx-\,\sum _{t=0}^{T-1}\int _{\Omega }\lambda _{t+1}(x) \int _{\Omega }\frac{1}{1+r_{t}(y)\frac{W^{*}(y)}{W_{t}(y)}}\frac{\partial f(W_{t}(y),y)}{\partial W} \psi _{t}(y) k(x,y)dydx\\&\quad =\sum _{t=0}^{T-1}\int _{\Omega } \lambda _{t+1}(x) \left[ \psi _{t+1}(x)-\int _{\Omega }\frac{1}{1+r_{t}(y)\frac{W^{*}(y)}{W_{t}(y)}} k(x,y)\frac{\partial f(W_{t}(y),y)}{\partial W} \psi _{t}(y)dy \right] dx\\&\quad =\sum _{t=0}^{T-1}\int _{\Omega } -\lambda _{t+1}(x)\int _{\Omega } \bigg (\frac{1}{1+r_{t}(y)\frac{W^{*}(y)}{W_{t}(y)}}\bigg )^{2} l_{t}(y) k(x,y)f(W_t (y),y)dy dx\\&\quad =\sum _{t=0}^{T-1}-\int _{\Omega } l_{t}(x)f(W_t (x),x) \bigg (\frac{1}{1+r_{t}(x)\frac{W^{*}(x)}{W_{t}(x)}}\bigg )^{2} \int _{\Omega } \lambda _{t+1}(y) k(y,x)dy dx \end{aligned}$$

where the third equality is obtained by switching the order of integration in the second integral. Going back to Eq. (24) we obtain:

25 $$\begin{aligned} \begin{aligned}&\sum _{t=0}^{T-1}-\int _{\Omega }\bigg [\bigg ( l_{t}(x)f(W_t (x),x)\bigg (\frac{1}{1+r_{t}(x)\frac{W^{*}(x)}{W_{t}(x)}}\bigg )^{2} \int _{\Omega } \lambda _{t+1}(y) k(y,x)dy\bigg )\\&\qquad +\,n_{t}W^{*}(x)l_{t}(x)+2s_{t}r_{t}^{*}l_{t}(x)(W^{*})_{2}(x)\bigg ] dx \end{aligned} \end{aligned}$$

We need

26 $$\begin{aligned} \begin{aligned}&- l_{t}(x)f(W_t (x),x)\bigg (\frac{1}{1+r_{t}(x)\frac{W^{*}(x)}{W_{t}(x)}}\bigg )^{2} \int _{\Omega } \lambda _{t+1}(y) k(y,x)dy+n_{t}W^{*}(x)l_{t}(x)\\&\qquad +\,2s_{t}r_{t}^{*}l_{t}(x)(W^{*})^{2}(x) =0 \end{aligned}\nonumber \\ \end{aligned}$$

(to ensure minimization of cost functional) from which we get

27 $$\begin{aligned}&-f(W_t (x),x)\bigg (\frac{1}{1+r_{t}(x)\frac{W^{*}(x)}{W_{t}(x)}}\bigg )^{2} \nonumber \\&\qquad \times \int _{\Omega } \lambda _{t+1}(y) k(y,x)dy+n_{t}W^{*}(x)+2s_{t}r_{t}^{*}(W^{*})^{2}(x) =0. \end{aligned}$$

Defining

28 $$\begin{aligned}&\displaystyle A_{1}=f(W_t (x),x)\int _{\Omega } \lambda _{t+1}(y) k(y,x)dy, \end{aligned}$$

29 $$\begin{aligned}&\displaystyle B=\frac{W^{*}(x)}{W_{t}(x)}, \end{aligned}$$

30 $$\begin{aligned}&\displaystyle C=n_{t}W^{*}(x), \end{aligned}$$

31 $$\begin{aligned}&\displaystyle D=2s_{t}(W^{*})^{2}(x), \end{aligned}$$

allows Eq. (27) to be written as:

32 $$\begin{aligned} \frac{-A_{1}}{(1+Br)^{2}}+C+Dr=0. \end{aligned}$$

We can transform Eq. (32) as:

33 $$\begin{aligned} A_{1}=\left( C-\frac{D}{B}\right) (1+Br)^2+\frac{D}{B}(1+Br)^{3}. \end{aligned}$$

Letting $X=1+Br$, $b=\frac{CB}{D}-1=\frac{n}{2sW}-1$ and $d=\frac{-A_{1}B}{D}=\frac{-f(W_t (x),x)\int _{\Omega } \lambda _{t+1}(y) k(y,x)dy}{2sW_{t}W^{*}}$ in Eq. (33) we obtain the following cubic equation:

34 $$\begin{aligned} X^{3}+bX^{2}+d=0. \end{aligned}$$
